# miR-1207-5p Can Contribute to Dysregulation of Inflammatory Response in COVID-19 *via* Targeting SARS-CoV-2 RNA

**DOI:** 10.3389/fcimb.2020.586592

**Published:** 2020-10-29

**Authors:** Giorgio Bertolazzi, Chiara Cipollina, Panayiotis V. Benos, Michele Tumminello, Claudia Coronnello

**Affiliations:** ^1^ Department of Economics, Business and Statistics, University of Palermo, Palermo, Italy; ^2^ Fondazione Ri.MED, Palermo, Italy; ^3^ Institute for Biomedical Research and Innovation, National Research Council, Palermo, Italy; ^4^ Department of Computational and Systems Biology, University of Pittsburgh School of Medicine, Pittsburgh, PA, United States

**Keywords:** microRNA regulatory network, SARS-CoV-2, macrophage recruitment, inflammatory response, competing RNAs, miRNA target prediction

## Abstract

The present study focuses on the role of human miRNAs in SARS-CoV-2 infection. An extensive analysis of human miRNA binding sites on the viral genome led to the identification of miR-1207-5p as potential regulator of the viral Spike protein. It is known that exogenous RNA can compete for miRNA targets of endogenous mRNAs leading to their overexpression. Our results suggest that SARS-CoV-2 virus can act as an exogenous competing RNA, facilitating the over-expression of its endogenous targets. Transcriptomic analysis of human alveolar and bronchial epithelial cells confirmed that the CSF1 gene, a known target of miR-1207-5p, is over-expressed following SARS-CoV-2 infection. CSF1 enhances macrophage recruitment and activation and its overexpression may contribute to the acute inflammatory response observed in severe COVID-19. In summary, our results indicate that dysregulation of miR-1207-5p-target genes during SARS-CoV-2 infection may contribute to uncontrolled inflammation in most severe COVID-19 cases.

## Introduction

COVID-19 is the first worldwide pandemic in a globalized world. The short time since the outbreak is the reason why many aspects of the molecular interactions of SARS-CoV-2 in the human host are still unknown, especially its mechanisms at transcriptional level. The present study aims to unravel the role of human miRNAs in SARS-CoV-2 infection. miRNAs are short non-coding RNA molecules with a post-transcriptional regulatory function (Bartel, 2004). They bind complementary sequences in mRNA molecules, with the role of inhibiting the translation of their mRNA targets into proteins (Bartel, 2009). Host endogenous miRNA activity in viral propagation has been previously studied and many complex virus-specific mechanisms have been identified, although the precise role of miRNAs in viral infections is not yet fully understood (Bruscella et al., 2017). In this paper, we present the results of an extensive predictive analysis to identify human lung-specific miRNAs that may bind the SARS-CoV-2 RNA. Then, we considered the already experimentally validated miRNA interactions with endogenous genes to identify the host’s miRNA regulatory sub-network affected by SARS-CoV-2 infection, looking at the virus as a competing RNA (Sumazin et al., 2011). We finally evaluated the impact of such interactions on the expression profile of genes targeted by the identified miRNAs in human airway epithelial cells infected with SARS-CoV-2. Specifically, we identified miR-1207-5p as a possible regulator of the S protein in SARS-CoV-2 RNA. As so, we suggest that the viral RNA competes with the CSF1 mRNA, a known target of miR-1207-5p (Dang et al., 2016), leading to CSF1 overexpression. To support our hypothesis, several published transcriptional datasets were evaluated. The finding that the CSF1 gene is over-expressed in lung epithelial cells infected with SARS-CoV-2 supported our hypothesis. CSF1 controls the production, differentiation and function of macrophages and its overexpression may contribute to the acute inflammatory response observed in severe COVID-19.

## Methods

### Transcriptomics Datasets and Analysis

Normal lung tissue expression profiles have been downloaded from TissueAtlas (Ludwig et al., 2016). Raw miRNA expression data from 18 lung control tissues were normalized with quantile normalization and the average expression level for each miRNA was computed. We used the average expression profile computed from all the 18 control tissues to identify the top 100 expressed miRNAs in normal lung tissue. **Table S1** summarizes the list of selected miRNAs and their average expression level in lung control tissues.

A wide collection of already available transcriptomics datasets with gene expression profiles after SARS-CoV-2 infection has been assembled from literature, as summarized in **Table S2**. When available, we considered the differential expression analysis results obtained by the authors. Otherwise, we preprocessed and analyzed the gene expression profiles as specified in column “Data analysis” of **Table S2**. When raw count RNAseq data was available, we used the DESeq2 (Love et al., 2014) R pipeline to compare infected *vs.* not infected samples, and the Benjamini-Hochberg procedure (Benjamini, 1995) to compute adjusted p-values. The univariate threshold of statistical significance was set at 5%.

### SARS-CoV-2 Sequences

The RefSeq sequence NC_045512 was used as reference to predict the binding sites of human miRNAs on the viral RNA. A total of 15881 worldwide viral complete genomes was downloaded—updated to September 7th, 2020—from the Severe acute respiratory syndrome coronavirus 2 data hub of NCBI Virus database, by filtering for taxid = “2697049” and Nucleotide Completeness = “complete”. Stability of particular viral genome regions was assessed by searching the exact match of the region in all the viral available genomes. To assess the statistical significance of the stability of each binding site, we associated a p-value with the number (m_bs_) of viral sequences that showed a mutation in the region of the binding site. Such a p-value was calculated as the frequency with which a number of mutations larger or equal to m_bs_ was observed in all of the other regions with the same length of the binding site in the involved mRNA.

### miRNA Target Prediction

Mature miRNA sequences were downloaded from miRbase, version 22. We used four miRNA target prediction tools to assess whether an RNA sequence is predicted to be a target of a miRNA: miRanda (Enright et al., 2003), PITA (Kertesz et al., 2007), Targetscan (Agarwal et al., 2015), and ComiR (Coronnello et al., 2012; Coronnello and Benos, 2013; Bertolazzi et al., 2020). miRanda script was used with -score 0 and -energy 0 settings. PITA and Targetscan scripts were used with default settings. ComiR was used to compute the ComiR score associated with the targets of each single miRNAs. For each miRNA we identified as highly predicted targets the genes that passed all the following conditions:

- miRanda binding energy, lower than -20;
- PITA ΔΔE, lower than -15
- TargetScan Binding Site, 8mer or 7mer
- ComiR score, greater than 0.85

We used the localization of the binding sites predicted by PITA, miRanda and Targetscan to further restrict the set of targets by considering only the binding sites predicted by all the three algorithms. The resulting targets are named as highly predicted targets.

Experimentally validated miRNA targets were downloaded from miRTarBase, where only the validation methods with strong evidence (i.e. Reporter assays, RT-qPCR, and Western-blot based experiments) have been considered.

## Results

### Five Human Lung-Specific miRNAs are Predicted to Target SARS-CoV-2 Viral Genome

**Figure 1:**
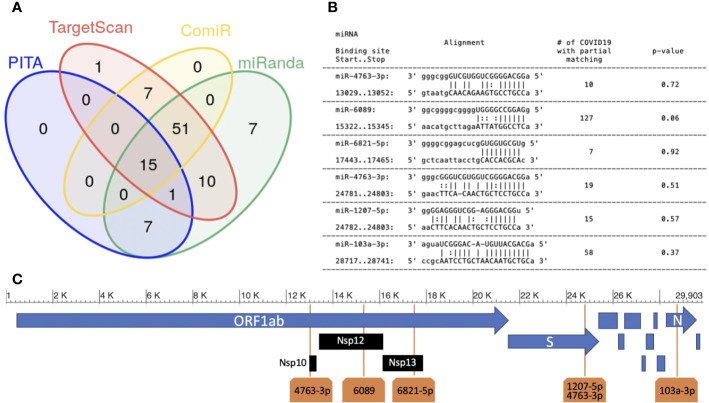
Human miRNAs targeting SARS-CoV-2 sequence. **(A)** miRNA target prediction results of the 100 most highly expressed miRNAs in normal lung on SARS-CoV-2 viral RNA (NCBI Reference sequence NC_045512.2). Each group in the Venn diagram represents the number of miRNAs with target(s) on the SARS-CoV-2 sequence (algorithms used: PITA, Targetscan, miRanda and ComiR); **(B)** six miRNA:viral-RNA targets predicted by all methods (“high confidence targets”). Column-1: miRNA name, start/stop bases in the NC_045512 sequence; column-2: base alignment; column-3: number of SARS-CoV-2 sequences not containing an exact match for the binding site region; column-4: p-value (see *Methods*); **(C)** the location of the five high confidence targets on the SARS-CoV-2 genome.

Aiming to unravel the role of endogenous miRNA expressed in the human lung with respect to SARS-CoV-2 virus, we focused our analysis on the 100 most expressed miRNAs in normal lung (Ludwig et al., 2016), identified as described in *Methods*. We identified potential targets of these 100 miRNAs on SARS-CoV-2 RNA sequence (NCBI reference viral sequence NC_045512), using four miRNA target prediction tools (Enright et al., 2003; Kertesz et al., 2007; Coronnello and Benos, 2013; Agarwal et al., 2015) (see Methods). Only 15 miRNAs were predicted to target the viral RNA by all the four algorithms (**Figure 1A**). Among the predicted miRNA:viral RNA interacting pairs (specific target locations), six were identified by all four algorithms (**Figures 1B, C**).

The six sites were targeted by 5 miRNAs: miR-6089, miR-6821-5p, miR-103a-3p, miR-4763-3p, and miR-1207-5p. miR-4763-3p and miR-1207-5p miRNAs belong to the same miRNA family, sharing the same seed sequence (ggcaggg). In our analysis, we predict that they have a common binding site in the viral sequence, located in the region coding for the Spike (S) glycoprotein. Spike is a structural protein that allows Sars-Cov-2 to enter host cells by interacting with membrane receptors (Masters, 2006). Human miRNAs miR-6089, miR-6821-5p, and miR-4763-3p have their binding sites in the ORF1ab gene, specifically hitting the regions coding for Nsp10, formerly known as growth-factor-like protein (GFL), Nsp12, an RNA-dependent RNA polymerase, and Nsp13_ZBD gene, a helicase (**Figure 1C**). The three mentioned non-structural proteins are crucial in coronavirus replication, being part of a complex of 16 non-structural proteins entailed for viral RNA replication and transcription (Masters, 2006; Bouvet et al., 2014). miR-103a-3p binding site is located in the Nucleocapsid (N) protein coding region. N proteins are structural proteins, that play key roles during the packaging of the viral RNA genome (Masters, 2006). Whether the enhancement of the host’s miRNAs regulatory machinery could inhibit the replication process or the production of the structural viral proteins, and as a consequence the virus diffusion through the host, is a hypothesis that needs to be experimentally validated and requires further investigation.

### Stability of Predicted miRNA Binding Sites on SARS-CoV-2 RNA

The worldwide spread of COVID-19 infection exposes the viral genome to a high risk of mutation. For this reason, we checked the binding sites’ sequence stability across the 15881 SARS-CoV-2 genomes annotated from all over the world in the NCBI virus database. To analyze such a stability, for each one of the six selected binding sites, we counted the number of viral sequences that presented a mutation. Results are reported in the third column of **Figure 1B**. We found that the binding regions are highly stable, which implies the consequent stability of binding-site predictions across the currently circulating viruses. In addition, we compared the occurrences of mutations in each binding site with the occurrences in any other region of the same length in the involved viral coding RNA, as described in the Methods section. The obtained p-values (see **Figure 1B**) indicate that the stability of all the six binding sites does not show a significant deviation from the one of the whole mRNA in which they are located, respectively.

### Host mRNAs Competing With SARS-CoV-2 RNA are Overexpressed in Lung Epithelial Cells

The viral sequence, once expressed, can interact with the host’s miRNA regulatory machine by sequestering the selected miRNAs. Therefore, viral RNA may act as a miRNA sponge, with the same mechanism of competing endogenous RNA (Sumazin et al., 2011). Among the five selected miRNAs, two have been previously studied in detail. miR-103a-3p activity has been widely studied in different tissues, i.e. gastric and colorectal cancer or liver (Liao and Lönnerdal, 2010; Martello et al., 2010; Annibali et al., 2012; Chen et al., 2012; Yu et al., 2012; Zhang et al., 2012; Geng et al., 2014; Liang et al., 2015; Zhang et al., 2015; Asiaee et al., 2019), and a number of its targets has been validated. miR-1207-5p expression is high in the cytoplasmic fraction of human normal lung tissue while being reduced in cancer (Dang et al., 2016). miR-1207-5p has been first characterized as negative regulator of epithelial-to-mesenchymal transition (EMT) as it inhibits the expression of a number of genes involved in this process, including Snail, Smad2, Smad3 and Vimentin (Dang et al., 2016; Qin et al., 2016). In addition to its role in EMT, miR-1207-5p plays an important role in shaping the inflammatory milieu. In this respect, CSF1 (colony-stimulating factor 1, also known as macrophage colony-stimulating factor, M-CSF) has been reported as one of its direct targets (Dang et al., 2016).

In order to test whether infection of lung epithelial cells with SARS-CoV-2 cell infection affects the gene expression levels of the endogenous miRNA target genes, we used a recent dataset of gene expression profiles of human lung-derived cells infected with SARS-CoV-2 (Blanco-Melo et al., 2020). Authors examined the behavior of wild type adenocarcinomic human alveolar basal epithelial (A549) and airway epithelial (Calu3) cell lines. A549 cells show a low expression of ACE2 receptor, hence a limited coronavirus infection rate. Thus, the authors also analyzed A549 cells transfected with a vector expressing ACE2 (A549+ACE2). We used this dataset to analyze the transcriptional profiling of the experimentally validated targets of miR-1207-5p and miR-103a-3p.

**Figure 2:**
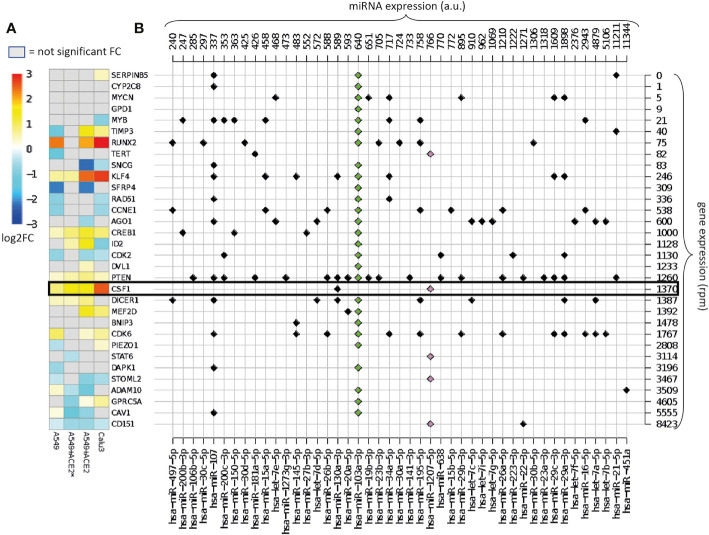
Overview of the validated targets of hsa-miR-1207-5p and hsa-miR-103a-3p. **(A)** Heatmap of the log2FC in gene expression between SARS-CoV-2 infection *vs.* mock treatment in cells with different multiplicities of infection (MOI). Cells: A549 (low ACE2 expression), A549+ACE2* (ACE2-expressing A549 cells, low MOI = 0.2), A549+ACE2 (ACE2-expressing A549 cells, MOI = 2–5), Calu3 cells (MOI = 2–5) (GEO dataset GSE147507). Only the log2FC that are associated with adjusted p-value <0.05 are displayed. Target genes are ordered according to their average expression level in A549 cells. **(B)** Map of annotated interactions among the targets of hsa-miR-1207-5p (pink) and hsa-miR-103a-3p (green) and other highly expressed miRNAs in normal lung tissue miRNAs. miRNAs are ordered according to their expression level, shown on the top of the grid. Genes are in the same order as in panel **(A)** and their expression levels are shown on the right of the grid.

**Figure 2A** presents the effect of viral infection on miR-1207-5p and miR-103a-3p endogenous target gene expression. Log-fold change (log2FC) values are calculated by comparing SARS-CoV-2 infected *vs.* mock treated cell lines (details in Methods). We expect that endogenous direct targets will increase their expression level following SARS-CoV-2 infection since viral RNA will compete with the endogenous RNA. Some of the analyzed targets behave as expected, especially the ones that are in the range of 1,000–2,000 reads per million (rpm), including CREB1, CSF1, PTEN, and DICER1. Consistent with the known A549 limited infection rate, the expression of these genes is enhanced in ACE2-expressing A549 cells, and even more in Calu-3 cells that are highly permissive to SARS-CoV-2 replication. These findings support our hypothesis that the viral RNA may act as a competing RNA for a selection of host miRNAs leading to the increase of the expression level of their endogenous targets.

Highly expressed targets, for instance ADAM10, are not up-regulated as expected. This is probably due to the fact that these genes might be modulated by other highly expressed miRNAs not sequestered by the virus. Alternatively, the sponge effect that we are hypothesizing is not effective when the mRNA is highly expressed.

**Figure 2B** presents the complexity of the miRNA-target network known up to now. Here we map all the experimentally validated interactions among the list of direct targets of miR-1207-5p and miR-103a-3p, and 45 of the 100 most highly expressed miRNAs in healthy lungs, that show at least one interaction. For instance, we observe that ADAM10, one of the targets of miR-103a-3p, is also regulated by miR-451a, the most highly expressed miRNA in lung. The presence of this regulator might be the reason why the expression of ADAM10 is not affected by the presence of the virus.

### Binding of miR-1207-5p to SARS-CoV-2 RNA May Lead to Over-Expression of EMT-Related Genes and CSF1

**Figure 3:**
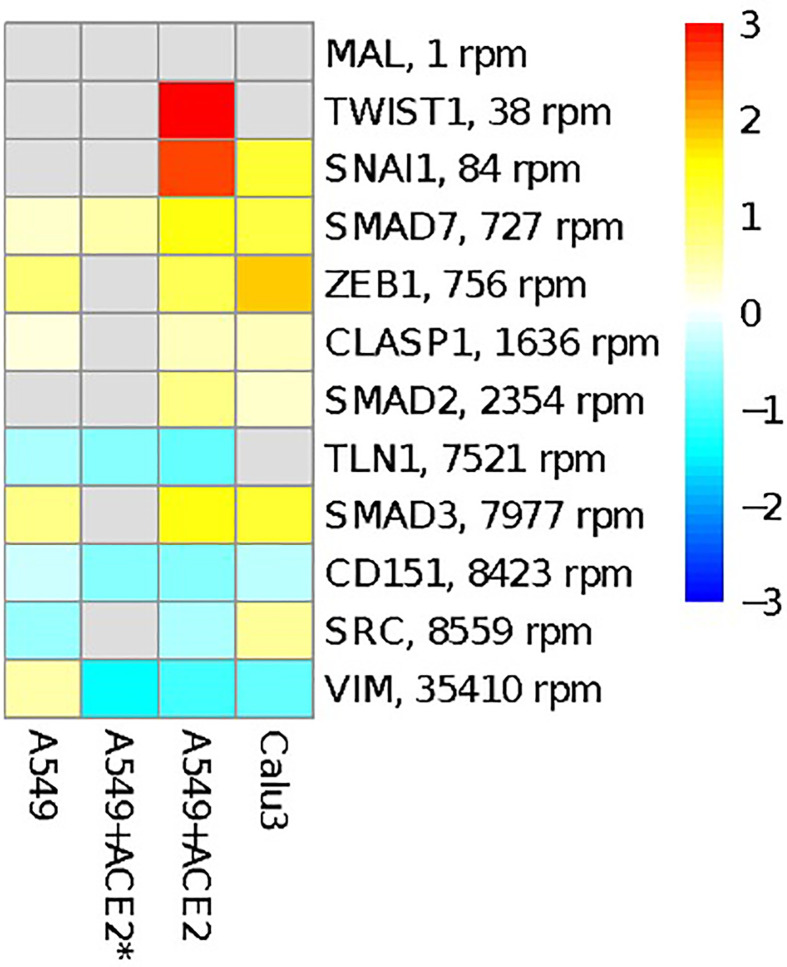
Overview of genes involved in EMT process and reported to be regulated by miR-1207-5p. The heatmap shows the log2FC in gene expression between SARS-CoV-2 infection *vs.* mock treatment in the same cells as in **Figure 2**. Targets are ordered according to their average expression level in A549 cells.

miR-1207-5p has been first characterized as negative regulator of EMT by controlling the expression of several genes including SMAD2, SMAD3, SMAD7, CLASP1, ZEB1, and SNAIL1 (Dang et al., 2016; Qin et al., 2016). EMT processes favor fibrotic events. Of interest, current data suggest that pulmonary fibrosis after COVID-19 recovery could be substantial (Cabrera-Benitez et al., 2014; Merad and Martin, 2020; Spagnolo et al., 2020). Therefore, we tested the hypothesis that SARS-CoV-2 infection in bronchial epithelial cells may have an impact on the expression of these genes by reducing the availability of miR-1207-5p. **Figure 3** shows the results of the differential expression analysis for the genes involved in EMT that have been reported to be regulated by miR-1207-5p. The increase in their expression levels appears evident when cells are infected with SARS-CoV-2 virus, therefore supporting our hypothesis.

We further expanded our analysis by evaluating the impact of SARS-CoV-2 infection on the expression of CSF1. As reported in **Figure 2**, CSF1 is one of the host gene targets most upregulated following viral infection. CSF1 is a predicted target of 3 out of 5 of the miRNAs targeting the virus sequence: miR-4763-3p, miR-1207-5p and miR-6089. It is also an experimentally validated target of miR-1207-5p (Dang et al., 2016). The only other known miRNA CSF1 regulator, among the 100 highly expressed miRNA in the lung, is miR-130a-3p, which is expressed at lower level than miR-1207-5p. CSF1 regulates the survival, proliferation, differentiation, and chemotaxis of tissue macrophages and dendritic cells (DC) that play a key role in innate immune responses. In the human lung, CSF1 can be released by airway epithelial cells in the airspace and its local concentration contributes to control the recruitment and activation of DC and macrophages (Louis et al., 2015; Moon et al., 2018; Turianová et al., 2019).

To further validate our hypothesis that the CSF1 mRNA is over-expressed after SARS-CoV-2 infection, we analyzed several recently published datasets as reported in **Table S2**. To this purpose, different types of experimental designs and platforms were taken into consideration. When available, we referred to the differential expression analysis performed by the authors. Specifically, we considered transcriptomics data analysis of infected *vs.* healthy samples from human lung biopsies as reported in (Vishnubalaji et al., 2020), bronchoalveolar lavage fluid (BALF) in (Zhou et al., 2020), peripheral blood mononuclear cells (PBMC) and BALF in (Xiong et al., 2020), and whole blood in (Ong et al., 2020). We also analyzed the single cell RNAseq data from whole blood reported in ref. (Wilk et al., 2020), infected NHBE cells in (Ravindra et al., 2020), and infected Calu3 cells in (Emanuel et al., 2020). Data sets obtained by analyzing human samples were not useful to confirm our hypothesis. This can be due to several reasons. More specifically, the high-variability among patients, the cell heterogeneity of reported biological samples (such as bronchioalveolar lavage fluids and lung biopsies) with different efficiency of viral transfection and the low sample size make it really difficult to unravel fine regulatory mechanisms of virus-host interaction. On the contrary, when dataset derived from bronchial epithelial cells (both primary cells and cell lines) were analyzed, significant upregulation of CSF1 was observed therefore confirming our hypothesis. For example, Wyler et al. (Emanuel et al., 2020) performed gene expression profiles of SARS-CoV-2 infected Calu3 cell line. Overexpression of CSF1 in Sars-CoV-2 infected versus mock treated cells confirmed our hypothesis. Furthermore, in Ravindra et al. (2020) the authors performed single-cell RNA sequencing of human bronchial epithelial cells grown in air-liquid interface and infected with SARS-CoV-2. When looking at ciliated cells, the expression of CSF1 significantly increased in infected compared to mock cells. Of note, the expression of CSF1 was significantly higher in ciliated infected cells compared to bystander cells that remained uninfected in samples challenged with SARS-CoV-2. These findings suggest that viral replication inside the cells is required in order for CSF-1 to be over-expressed therefore supporting that a direct interaction between viral RNA and host miRNAs is required to alter the expression of CSF1 during infection.

## Discussion

In 10–20% of the cases, SARS-CoV-2 infections may progress to interstitial pneumonia and acute respiratory distress syndrome (ARDS) especially in patients with older age and comorbidities. Clinical features of severe COVID-19 as well as their systemic cytokine profile suggest the occurrence of macrophage activation syndrome (MAS) (McGonagle et al., 2020; Merad and Martin, 2020). High rates of viral replication have been listed among the factors that may drive severe lung pathology during infection by contributing to enhanced host cell cytolysis and production of inflammatory cytokines and chemokines by infected epithelial cells (Merad and Martin, 2020; Wen et al., 2020; Gardinassi et al., 2020). We propose that the high concentration of viral RNA in the cell may sequestrate miR-1207-5p therefore contributing to CSF1 release leading to enhanced macrophage recruitment and activation. In fact, increased release of CSF1 may represent a predisposing factor for MAS and cytokine storm secondary to viral infection (Akashi et al., 1994; Maruyama and Inokuma, 2010). Consistently, it has been recently reported that T-cell derived CSF-1, acting *via* intercellular crosstalk, may be associated with cytokine storm in COVID-19 (Wen et al., 2020). In our proposed model, infected bronchial epithelial cells may be a source of CSF-1 contributing to local and systemic inflammatory profiles. In addition, reduced availability of miR-1207-5p may also promote EMT events therefore favoring fibrosis (Cabrera-Benitez et al., 2014; Merad and Martin, 2020; Spagnolo et al., 2020). Although further experimental validation will be required to confirm direct interaction between miR-1207-5p and the SARS-CoV-2 genome, our proposed model has been confirmed using several published datasets. Results herein reported strongly suggest that upregulation of CSF1 due to interaction of miR-1207-5p with viral genome may occur when lung epithelial cells are infected with a high viral load. A limitation of the current study is the lack of data regarding protein levels and release. To address this issue, we carefully looked for published proteomics data in COVID19 literature, but, so far, no information about CSF1 protein levels has been published and therefore further studies will be carried out to address this point. Nevertheless, transcriptional and post-transcriptional control of mRNA levels represent a key regulatory step for most inflammatory mediators during infection. In this respect, the discovery of novel potential mechanisms that contribute to modulate the mRNA levels of a specific inflammatory mediator in the context of SARS-Cov-2 infection may represent a step forward toward a better understanding of virus-host interaction molecular mechanisms.

A wide analysis of the SARS-CoV-2 transcriptome (Kim et al., 2020) revealed the presence of several non-canonical sub-genomic RNAs. They consist in discontinuous transcriptions of the viral sequence, where the 5’ leader region is fused to a non-conventional part of the genome. As a result, the obtained RNA contains only a portion of the viral mRNAs. It is tempting to speculate that they may play a role as competing RNA. Specifically, miR-1207-5p related binding site is located in the far downstream region of the viral gene Spike. As a consequence, almost all of the sub-genomic RNA sequences with the fusion occurring in the region of the Spike gene contain the miR-1207-5p binding site. Although, these sub-genomic RNA sequences do not have the coding potential to yield the S protein, they could still act as miRNA sponges.

To conclude, our results suggest that the miR-1207-5p family may interact with SARS-CoV-2 viral genome leading to deregulation of CSF-1, which may enhance inflammatory responses in COVID-19 patients, and promoting EMT, which can contribute to pulmonary fibrosis, a possible sequela of COVID-19. Further experimental validation will be conducted to confirm molecular mechanisms of host-virus interaction and to investigate their involvement in disease progression.

## Data Availability Statement

The original contributions presented in the study are included in the article/**Supplementary Materials**. Further inquiries can be directed to the corresponding authors.

## Author Contributions

Conceptualization, MT, CCi, and CCo. Methodology, MT and CCo. Formal analysis, GB, MT, and CCo. Investigation, CCo. Writing—original draft, CCo. Writing—review and editing, GB, PB, CCi, MT, and CCo. Supervision, MT and CCo. All authors contributed to the article and approved the submitted version.

## Funding

The present work has been funded by Regione Siciliana, Assessorato delle Attività Produttive, Azione 1.1.5 del PO FESR Sicilia 2014/2020, Project n. 086202000366 – “OBIND”, CUP G29J18000700007 to MT and CC and by the National Institutes of Health (NIH) Grants U01HL137159 and R01LM012087 to PVB.

## Conflict of Interest

The authors declare that the research was conducted in the absence of any commercial or financial relationships that could be construed as a potential conflict of interest.

## References

[B1] Agarwal V. Bell G. W. Nam J. W. Bartel D. P. (2015). Predicting effective microRNA target sites in mammalian mRNAs. Elife 4, e05005. 10.7554/eLife.05005 PMC4532895 26267216

[B2] Akashi K. Hayashi S. Gondo H. Mizuno S. Harada M. Tamura K. (1994). Involvement of interferon-γ and macrophage colony-stimulating factor in pathogenesis of haemophagocytic lymphohistiocytosis in adults. Br. J. Haematol 87 (2), 243–250. 10.1111/j.1365-2141.1994.tb04905.x 7947264

[B3] Annibali D. Gioia U. Savino M. Laneve P. Caffarelli E. Nasi S. (2012). A new module in neural differentiation control: Two microRNAs upregulated by retinoic acid, miR-9 and -103, target the differentiation inhibitor ID2. PLoS One 7 (7), e40269. 10.1371/journal.pone.0040269 22848373 PMC3405103

[B4] Asiaee A. Abrams Z. B. Nakayiza S. Sampath D. Coombes K. R. (2019). Explaining Gene Expression Using Twenty-One MicroRNAs. J. Comput. Biol 27 (7), 1157–1170. 10.1089/cmb.2019.0321 31794247 PMC7398443

[B5] Bartel D. P. (2004). MicroRNAs: Genomics, Biogenesis, Mechanism, and Function. Cell 116 (2), 281–297. 10.1016/S0092-8674(04)00045-5 14744438

[B6] Bartel D. P. (2009). MicroRNAs: Target Recognition and Regulatory Functions. Cell 136 (2), 215–233. 10.1016/j.cell.2009.01.002 19167326 PMC3794896

[B7] Benjamini Y. Hochberg Y. (1995). Controlling the False Discovery Rate - a Practical and Powerful Approach to Multiple Testing. Journal of the Royal Statistical Society Series B-Methodological. J. R. Stat. Soc. Ser. B (Methological) 57 (1), 289–300. 10.1111/j.2517-6161.1995.tb02031.x

[B8] Bertolazzi G. Benos P.V. Tumminello M. Coronnello C. (2020). An improvement of ComiR algorithm for microRNA target prediction by exploiting coding region sequences of mRNAs. BMC Bioinformatics 21, 201. 10.1186/s12859-020-3519-5 32938407 PMC7493982

[B9] Blanco-Melo D. Nilsson-Payant B. E. Liu W. C. Uhl S. Hoagland D. Moller R. (2020). Imbalanced Host Response to SARS-CoV-2 Drives Development of COVID-19. Cell 181 (5), 1036–1045. 10.1016/j.cell.2020.04.026 32416070 PMC7227586

[B10] Bouvet M. Lugari A. Posthuma C. C. Zevenhoven J. C. Bernard S. Betzi S. (2014). Coronavirus Nsp10, a critical co-factor for activation of multiple replicative enzymes. J. Biol. Chem 289, 25783–25796. 10.1074/jbc.M114.577353 25074927 PMC4162180

[B11] Bruscella P. Bottini S. Baudesson C. Pawlotsky J. M. Feray C. Trabucchi M. (2017). Viruses and miRNAs: More friends than foes. Front. Microbiol 8, 824. 10.3389/fmicb.2017.00824 28555130 PMC5430039

[B12] Cabrera-Benitez N. E. Laffey J. G. Parotto M. Spieth P. M. Villar J. Zhang H. (2014). Mechanical ventilation-associated lung fibrosis in acute respiratory distress syndrome: A significant contributor to poor outcome. Anesthesiology 121, 189–198. 10.1097/ALN.0000000000000264 24732023 PMC4991945

[B13] Chen H. Y. Lin Y. M. Chung H. C. Lang Y.D. Lin C. J. Huang J. (2012). MiR-103/107 promote metastasis of colorectal cancer by targeting the metastasis suppressors DAPK and KLF4. Cancer Res. 10.1158/0008-5472.CAN-12-0667 22593189

[B14] Coronnello C. Hartmaier R. Arora A. Huleihel L. Pandit K. V. Bais A. S. (2012). Novel modeling of combinatorial miRNA targeting identifies SNP with potential role in bone density, PLoS Comput. Biol. 8 (12), e1002830. 10.1371/journal.pcbi.1002830 23284279 PMC3527281

[B15] Coronnello C. Benos P. V. (2013). ComiR: Combinatorial microRNA target prediction tool. Nucleic Acids Res 41 (W1), W159–W164. 10.1093/nar/gkt379 23703208 PMC3692082

[B16] Dang W. Qin Z. Fan S. Wen Q. Lu Y. Wang J. (2016). miR-1207-5p suppresses lung cancer growth and metastasis by targeting CSF1. Oncotarget 7, 32421–32432. 10.18632/oncotarget.8718 27107415 PMC5078023

[B17] Emanuel W. Mosbauer K. Franke V. Diag A. Gottula L. T. Arsie R. (2020). Bulk and single-cell gene expression profiling of SARS-CoV-2 infected human cell lines identifies molecular targets for therapeutic intervention. bioRxiv. 10.1101/2020.05.05.079194

[B18] Enright A. J. John B. Gaul U. Tuschl T. Sander C. Marks D. S. (2003). MicroRNA targets in Drosophila. Genome Biol 5, R1. 10.1186/gb-2003-5-1-r1 14709173 PMC395733

[B19] Gardinassi L. G. Souza C. O. S. Sales-Campos H. Fonseca S. G. (2020). Immune and Metabolic Signatures of COVID-19 Revealed by Transcriptomics Data Reuse. Front. Immunol 11, 1636. 10.3389/fimmu.2020.01636 32670298 PMC7332781

[B20] Geng L. Sun B. Gao B. Wang Z. Quan C. Wei F. (2014). MicroRNA-103 promotes colorectal cancer by targeting tumor suppressor DICER and PTEN. Int. J. Mol. Sci 15 (5), 8458–8472. 10.3390/ijms15058458 24828205 PMC4057742

[B21] Kertesz M. Iovino N. Unnerstall U. Gaul U. Segal E. (2007). The role of site accessibility in microRNA target recognition. Nat. Genet 39, 1278–1284. 10.1038/ng2135 17893677

[B22] Kim D. Lee J. Y. Yang J. S. Kim J. W. Kim V. N. Chang H. (2020). The Architecture of SARS-CoV-2 Transcriptome. Cell 181 (4), 914–921. 10.1016/j.cell.2020.04.011 32330414 PMC7179501

[B23] Liang J. Liu X. Xue H. Qiu B. Wei B. Sun K. (2015). MicroRNA-103a inhibits gastric cancer cell proliferation, migration and invasion by targeting c-Myb. Cell Prolif 48 (1), 78–85. 10.1111/cpr.12159 25530421 PMC6496034

[B24] Liao Y. Lönnerdal B. (2010). Global MicroRNA characterization reveals that miR-103 is involved in IGF-1 stimulated mouse intestinal cell proliferation. PLoS One 5 (9), e12976. 10.1371/journal.pone.0012976 20886090 PMC2944884

[B25] Louis C. Cook A. D. Lacey D. Fleetwood A. J. Vlahos R. Anderson G. P. (2015). Specific Contributions of CSF-1 and GM-CSF to the Dynamics of the Mononuclear Phagocyte System. J. Immunol 195 (1), 134–144. 10.4049/jimmunol.1500369 26019271

[B26] Love M. II Huber W. Anders S. (2014). Moderated estimation of fold change and dispersion for RNA-seq data with DESeq2. Genome Biol 15, 550. 10.1186/s13059-014-0550-8 25516281 PMC4302049

[B27] Ludwig N. Leidinger P. Becker K. Backes C. Fehlmann T. Pallasch C. (2016). Distribution of miRNA expression across human tissues. Nucleic Acids Res 44 (8), 3865–3877. 10.1093/nar/gkw116 26921406 PMC4856985

[B28] Martello G. Rosato A. Ferrari F. Manfrin A. Cordenonsi M. Dupont S. (2010). A microRNA targeting dicer for metastasis control. Cell 141 (7), 1195–1207. 10.1016/j.cell.2010.05.017 20603000

[B29] Maruyama J. Inokuma S. (2010). Cytokine profiles of macrophage activation syndrome associated with rheumatic diseases. J. Rheumatol 37 (5), 967–973. 10.3899/jrheum.090662 20231207

[B30] Masters P. S. (2006). The Molecular Biology of Coronaviruses. Adv. Virus Res 66, 193–292. 10.1016/S0065-3527(06)66005-3 16877062 PMC7112330

[B31] McGonagle D. O’Donnell J. S. Sharif K. Emery P. Bridgewood C. (2020). Immune mechanisms of pulmonary intravascular coagulopathy in COVID-19 pneumonia. Lancet Rheumatol 2 (7), E437–E445. 10.1016/S2665-9913(20)30121-1 32835247 PMC7252093

[B32] Merad M. Martin J. C. (2020). Pathological inflammation in patients with COVID-19: a key role for monocytes and macrophages. Nat. Rev. Immunol 20, 355–362. 10.1038/s41577-020-0331-4 32376901 PMC7201395

[B33] Moon H. G. Kim S. Jeong J. J. Han S. S. Jarjour N. N. Lee H. (2018). Airway Epithelial Cell-Derived Colony Stimulating Factor-1 Promotes Allergen Sensitization. Immunity 49 (2), 275–287. 10.1016/j.immuni.2018.06.009 30054206 PMC6594049

[B34] Ong E. Z. Chan Y. F. Z. Leong W. Y. Lee N. M. Y. Kalimuddin S. Mohideen S. M. H. (2020). A Dynamic Immune Response Shapes COVID-19 Progression. Cell Host Microbe 27 (6), 879–882. 10.1016/j.chom.2020.03.021 32359396 PMC7192089

[B35] Qin Z. He W. Tang J. Ye Q. Dang W. Lu Y. (2016). MicroRNAs Provide Feedback Regulation of Epithelial-Mesenchymal Transition Induced by Growth Factors. J. Cell. Physiol 231 (1), 120–129. 10.1002/jcp.25060 26032086

[B36] Ravindra N. Alfajaro M. M. Gasque V. Habet V. Wei J. Filler R. B. (2020). Single-cell longitudinal analysis of SARS-CoV-2 infection in human airway epithelium. BioRxiv Prepr Serv Biol. 10.1101/2020.05.06.081695 PMC8007021 33730024

[B37] Spagnolo P. Balestro E. Aliberti S. Cocconcelli E. Biondini D. Della Casa G. (2020). Pulmonary fibrosis secondary to COVID-19: a call to arms? Lancet Respir. Med 8 (8), 750–752. 10.1016/s2213-2600(20)30222-8 32422177 PMC7228737

[B38] Sumazin P. Yang X. Chiu H. S. Chung W. J. Iyer A. Llobet-Navas D. (2011). An extensive MicroRNA-mediated network of RNA-RNA interactions regulates established oncogenic pathways in glioblastoma. Cell 147 (2), 370–381. 10.1016/j.cell.2011.09.041 22000015 PMC3214599

[B39] Turianová L. Lachová V. Svetlíkova D. Kostrábová A. Betáková T. (2019). Comparison of cytokine profiles induced by nonlethal and lethal doses of influenza A virus in mice. Exp. Ther. Med 18 (6), 4397–4405. 10.3892/etm.2019.8096 31777543 PMC6862669

[B40] Vishnubalaji R. Shaath H. Alajez N. M. (2020). Protein coding and long noncoding RNA (lncRNA)) transcriptional landscape in SARS-CoV-2 infected bronchial epithelial cells highlight a role for interferon and inflammatory response. Genes (Basel) 11 (7), 760. 10.3390/genes11070760 PMC7397219 32646047

[B41] Wen W. Su W. Tang H. Le X. Zhang X. Zheng Y. (2020). Immune cell profiling of COVID-19 patients in the recovery stage by single-cell sequencing. Cell Discovery 6, 31. 10.1038/s41421-020-0168-9 32377375 PMC7197635

[B42] Wilk A. J. Rustagi A. Zhao N. Q. Roque J. Martínez-Colón G. J. McKechnie J. L. (2020). A single-cell atlas of the peripheral immune response in patients with severe COVID-19. Nat. Med 26, 1070–1076. 10.1038/s41591-020-0944-y 32514174 PMC7382903

[B43] Xiong Y. Liu Y. Cao L. Wang D. Guo M. Jiang A. (2020). Transcriptomic characteristics of bronchoalveolar lavage fluid and peripheral blood mononuclear cells in COVID-19 patients. Emerg. Microbes Infect 9 (1), 761–770. 10.1080/22221751.2020.1747363 32228226 PMC7170362

[B44] Yu D. Zhou H. Xun Q. Xu X. Ling J. Hu Y. (2012). microRNA-103 regulates the growth and invasion of endometrial cancer cells through the downregulation of tissue inhibitor of metalloproteinase 3. Oncol. Lett. 3 (6), 1221–1226. 10.3892/ol.2012.638 22783422 PMC3392565

[B45] Zhang S. Y. Surapureddi S. Coulter S. Ferguson S. S. Goldstein J. A. (2012). Human CYP2C8 is post-transcriptionally regulated by microRNAs 103 and 107 in human liver. Mol. Pharmacol 82 (3), 529–540. 10.1124/mol.112.078386 22723340 PMC3422698

[B46] Zhang Y. Qu X. Li C. Fan Y. Che X. Wang X. (2015). miR-103/107 modulates multidrug resistance in human gastric carcinoma by downregulating Cav-1. Tumor Biol 36, 2277–2285. 10.1007/s13277-014-2835-7 25407491

[B47] Zhou Z. Ren L. Zhang L. Zhong J. Xiao Y. Jia Z. (2020). Heightened Innate Immune Responses in the Respiratory Tract of COVID-19 Patients. Cell Host Microbe 27 (6), 883–890. 10.1016/j.chom.2020.04.017 32407669 PMC7196896

